# A structural interpretation of the effect of GC-content on efficiency of RNA interference

**DOI:** 10.1186/1471-2105-10-S1-S33

**Published:** 2009-01-30

**Authors:** Chi Yu Chan, C Steven Carmack, Dang D Long, Anil Maliyekkel, Yu Shao, Igor B Roninson, Ye Ding

**Affiliations:** 1 Wadsworth Center, New York State Department of Health, 150 New Scotland Avenue, Albany, NY 12208, USA; 2 Cancer Center, Ordway Research Institute, Albany, New York 12208, USA; 3 Department of Molecular Genetics, University of Illinois at Chicago, Chicago, IL 60607, USA

## Abstract

**Background:**

RNA interference (RNAi) mediated by small interfering RNAs (siRNAs) or short hairpin RNAs (shRNAs) has become a powerful technique for eukaryotic gene knockdown. siRNA GC-content negatively correlates with RNAi efficiency, and it is of interest to have a convincing mechanistic interpretation of this observation. We here examine this issue by considering the secondary structures for both the target messenger RNA (mRNA) and the siRNA guide strand.

**Results:**

By analyzing a unique homogeneous data set of 101 shRNAs targeted to 100 endogenous human genes, we find that: 1) target site accessibility is more important than GC-content for efficient RNAi; 2) there is an appreciable negative correlation between GC-content and RNAi activity; 3) for the predicted structure of the siRNA guide strand, there is a lack of correlation between RNAi activity and either the stability or the number of free dangling nucleotides at an end of the structure; 4) there is a high correlation between target site accessibility and GC-content. For a set of representative structural RNAs, the GC content of 62.6% for paired bases is significantly higher than the GC content of 38.7% for unpaired bases. Thus, for a structured RNA, a region with higher GC content is likely to have more stable secondary structure. Furthermore, by partial correlation analysis, the correlation for GC-content is almost completely diminished, when the effect of target accessibility is controlled.

**Conclusion:**

These findings provide a target-structure-based interpretation and mechanistic insight for the effect of GC-content on RNAi efficiency.

## Background

RNA interference (RNAi) [1] is a sequence-specific gene silencing mechanism that can be mediated either by small interfering RNAs (siRNAs) of about 21 nt with two-nucleotide 3' overhang [2], or by stably expressed short hairpin RNAs (shRNAs), which are processed by Dicer into siRNAs [3,4]. The antisense (guide) strand guides Argonaute2 (Ago2), the catalytic component of the RNA-induced silencing complex (RISC), to cleave mRNA by base-pairing with the complementary site in the target. Large variation in the efficiency of siRNAs has been commonly observed [5]. Usually, only a small proportion of randomly selected siRNAs are potent. Thus, there has been a great interest in determining rules for improvement of RNAi design. It has been commonly observed that high GC content negatively correlates with RNAi activity. Thus, a low GC-content is among a number of empirical rules on siRNA duplex features that have been proposed [6]. In addition, the importance of target secondary structure and accessibility has been supported by numerous studies [7-14].

It is tempting to seek a mechanistic interpretation for the effect of GC-content on RNAi efficiency. Because high GC can give rise to stable RNA secondary structure, one possible interpretation is the proposal that self-structure of the siRNA guide strand can be detrimental to RNAi activity [15]. We here investigate this issue by considering the secondary structures for both the target messenger RNA (mRNA) and the siRNA guide strand. From analyses of a unique homogeneous data set of 101 shRNAs targeted to 100 endogenous human genes, the results support a target-structure-based interpretation for the effect of GC-content.

## Results

For the shRNA dataset, we first computed Pearson's correlation coefficient and the significance of the correlation for the RNAi activity and a structural parameter or GC%. These calculations were performed by using the R statistical package [16], and the results were summarized in Table 1. We find that, with the highest and significant correlation, target site accessibility is more important than GC-content. We also observe an appreciable negative correlation of -0.1444 between GC-content and RNAi activity, albeit with a *p*-value of 0.1497. Surprisingly, for the predicted optimal structure for the siRNA guide strand, there is a lack of correlation between RNAi activity and either the stability (siRNA MFE in Table 1) or the number of free dangling nucleotides at an end of the structure.

**Table 1:** Correlation analyses for structural parameters, GC%, and RNAi activity

Pair of measures		Pearson's correlation coefficient	Significance of correlation (*p*-value)
Δ*G*_disruption_	RNAi activity	0.2382	0.0165

siRNA GC%	RNAi activity	-0.1444	0.1497

siRNA MFE	RNAi activity	0.0531	0.5982

Number of 3' free nts	RNAi activity	0.0108	0.9146

Number of 5' free nts	RNAi activity	0.0538	0.5933

Number of 3' free nts with ≥ 2 5' free nts	RNAi activity	0.1311	0.2465

Δ*G*_disruption_	siRNA GC%	-0.6832	3.519E-15

**Figure 1:**
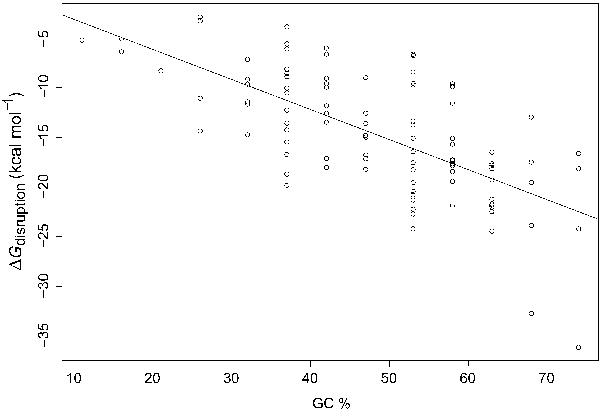
Strong correlation between Δ*G*_disruption _and GC-content of siRNA guide strand (linear regression line is shown for predicting Δ*G*_disruption _by GC%, with *R*^2 ^= 0.4668 and *P*-value = 3.52E-15).

Because the effect of GC-content cannot be explained by the structure of the siRNA guide strand, we hypothesized that, to some extent, GC-content is indicative of target site accessibility. Indeed, there is a highly significant correlation between target site accessibility and GC-content (Table 1; Figure 1). Furthermore, for the representative set of structural RNAs, 62.6% of paired bases are GC, significantly higher than 38.7% for unpaired bases (*p*-value = 5.67E-15 for the Wilcoxon signed rank test). Thus, for a structured RNA, a region with higher GC content is likely to have more stable secondary structure.

To further investigate the relationships among GC-content, target accessibility and RNAi activity, we performed partial correlation analysis. Partial correlation measures the degree of association between two random variables, with the effect of a set of controlling random variables removed. For the three variables of our interest here, the calculation was performed with a published R script for three variables [17]. By partial correlation analysis, we found that when the effect of target accessibility is controlled (i.e., removed), the correlation between GC-content and RNAi activity is 0.026, with a *p*-value of 0.7978. This near complete diminishment of correlation supports the hypothesis that the negative correlation typically observed between GC-content and RNAi efficiency is mainly due to structural inaccessibility often associated with high GC-content of the target site.

Because the protein complexes involved in gene regulation are similar for microRNAs [18] and siRNAs, we folded all of 137 worm microRNAs from microRNA Registry [19]. We found that for 79 (58%) of the microRNAs, the predicted optimal structure has a stability (i.e., free energy) under 0.0 kcal/mol, typically with two to five consecutive base pairs. Furthermore, for 9 (7%) of the microRNAs, either the 5' end or the 3' end is completely involved in intramolecular base-pairing. These suggest that some intramolecular structures can be tolerated for the regulatory functions by animal microRNAs, and these structures are likely to be weakened or completely abolished upon interaction with the RISC.

## Discussion

The results of the analyses suggest that, to a large extent, the effect of GC-content on RNAi is due to the target structure and site accessibility rather than the structure of the siRNA guide strand. However, for the purpose of the rational design of RNAi experiments, GC-content cannot be a substitute for predicted target site accessibility, owning to its substantially lower correlation (Table 1). Similar observations were made in a previous analysis of other RNAi datasets based on alternative structural calculations [9]. The common findings from this study and the previous study support a target-structure-based interpretation for the effect of GC-content on the RNAi efficiency.

Contrasting with the conclusion from a published study [15], we did not observe any effect of potential folding of siRNA guide strand on RNAi efficiency. It is likely that the ability for the siRNA guide strand to fold may be negatively affected by the enzymatic activity by the RNAi machinery including duplex unwinding by helicase. In addition, constrained by RISC, the siRNA guide strand is unlikely to fold freely into a stable structure, regardless of GC-content.

Based on nine siRNAs with 11 common nts for a region of a single target, it was reported that the RNAi activity was strongly correlated with the number of free (unpaired) dangling nts at the ends of the structure predicted for the siRNA guide strand, and there was a poor correlation for other sequence features and target accessibility [15]. With our much larger and more representative dataset, we did not observe any correlation for the number of free dangling nts. For the negative finding on target accessibility, there may be two reasons. First, the accessibility was calculated with probabilities of unpaired individual bases in the Boltzmann ensemble of RNA structures [20]. Although this represents a major improvement over the use of a single structure such as the optimal fold, the accessibility can be arguably better assessed by consideration of free energy changes for siRNA-target hybridization [7]. Second, the assessment of the effect of target accessibility on target recognition and RNAi activity requires controlling for the upstream effect of siRNA duplex asymmetry [7]. The small data set of nine siRNAs is inadequate. The analysis of 137 worm microRNAs suggests that some intramolecular structures can be tolerated for the regulatory functions by microRNAs, and these structures are likely to be weakened or completely abolished upon interaction with the RISC. Target cleavage by RNAi machinery and translation repression by microRNA pathway may have different effects on the structure of the target and the small RNA:target duplex. It is possible that the effects of intramolecular structure can be different between siRNAs and microRNAs, so that the lack of negative effect of structure for siRNAs cannot be simply reasoned by the lack of self-folding effect for microRNAs. Nevertheless, the results of our analyses on the shRNA data do not support the previous conclusion on the significance of the effects of structures of the siRNA guide strands [15].

## Conclusion

Target accessibility as primarily determined by target secondary structure is an important determinant for RNAi potency. The commonly observed negative effect of high siRNA GC-content on RNAi potency is due to generally poor target accessibility for a high GC target site, rather than the likelihood that the high GC siRNA guide strand may form stable intramolecular secondary structure. These findings provide a target-structure-based interpretation and mechanistic insight for the effect of siRNA GC-content on RNAi efficiency.

## Methods

### Short hairpin data set

We used a data set of 101 shRNA sequences targeting 100 different endogenous human genes (i.e., two shRNAs for only one gene). They were obtained from the analysis of a library of shRNA sequences generated from randomly fragmented cDNA of normalized (reduced-redundancy) cDNA of all of the genes expressed in the MCF-7 human breast carcinoma cells. The generation and testing of the library will be described in detail elsewhere (Maliyekkel, A., Shao, Y., Warholic, N., Cole, K., Ding, Y., and Roninson, I.B., in preparation). shRNA activity was determined by measuring the levels of each target mRNA by real-time PCR, in triplicate. Percent knockdown was calculated from the ratio of mRNA levels with and without doxycycline. This data set (see Additional file 1) was previously employed to assess the effect of target structure on RNAi efficiency [7], and is provided. The siRNAs resulting from shRNA cleavage by Dicer are mostly 19 bp or 20 bp in length (with additional 2-nt 3' overhang), at comparable yields [21]. Because the computational results are highly similar for both lengths, we here report the results for the length of 19 bp.

For several reasons, we chose not to consider other RNAi datasets available in the literature. For example, the large dataset from a Norvatis study [22] was based on a reporter assay for 34 genes, whereas the assay of our dataset measures RNAi activity in an endogenous context for a much larger number of human genes. The heavy overlapping between target sites for several published siRNA datasets [6,23] can introduce a bias in analysis. Because such bias is difficult to assess, we decided to avoid such problem in data selection in our earlier study [7].

### A target-structure based energetic parameter for assessment of target accessibility

A number of approaches have been published for quantifying target site accessibility for rational design of RNA-targeting nucleic acids. Based on target structures predicted by RNA folding algorithms, these methods are either probabilistic or energetic. Probabilistic methods assess the probability that a base or a block of bases is single stranded [20,24,25], whereas energetic methods model the energy exchanges of the hybridization process [7,26-31], thus arguably providing more refined measures of accessibility. For example, consider two target sites with (nearly) equal probability of being single stranded. If one site has high AU, and the other has high GC, then the energetic costs for disrupting the target structure, and the stabilities of the hybrid could be quite different for the two sites. In data analysis for some of our studies, energy measures were observed to give improved correlations than probabilistic measures. Thus, our efforts in recent years have focused on energetic models. Below, we briefly discuss several major methods.

The Sfold structure sample [32,33] allows computation of both probabilistic measures [24] and energetic measures of target accessibility [7,28-31]. It is well established that a single-stranded block of 4–5 nts can facilitate the nucleation step of the hybridization [34,35]. Thus, a moderate structure sample is sufficient for revealing potential effective sites by using block size of 4 nts for accessibility profiling [24]. The major advantage of using the structure sampling algorithm is that the time consuming partition-function calculation for the whole target sequence only needs to be computed once. Folding constraints such as maximum nucleotide distance *L *for two bases to form a pair can be imposed for "local" folding. Such local folding was found to be significant for prokaryotic applications [29]. For prokaryotes, transcription and translation are tightly coupled events so that the target mRNA is unlikely to be able to fold globally. In contrast, eukaryotic mRNAs are first transcribed in nucleus and then transported to cytoplasm where they can conceivably fold globally before they engage in interactions with other molecules in the cytoplasm for regulation of gene translation. Global folding using Sfold sampling algorithm can reveal highly unstructured sites that are well "conserved" in the likely mRNA structure population. These well-predicted sites can be valuable for the selection of effective target sites.

Target site disruption energy, Δ*G*_disruption_, is the energy cost of local disruption of the mRNA structure so that the binding site becomes completely single-stranded [7]. A largely single-stranded (i.e., structurally accessible) site does not require substantial structure alteration for the guide siRNA strand to bind to the target. Δ*G*_disruption _is a quantitative measure of the structural accessibility at the target site, and is calculated based on target secondary structures predicted by Sfold [32] to address the likely population of mRNA structures.

An alternative to the local disruption assumption is the global disruption model. For this model, as a result of siRNA:mRNA hybridization, the base pairs outside the target site can be rearranged so that the mRNA adopts a new globally altered structure. In this case, Δ*G*_after _must be calculated by refolding the mRNA with the binding site constrained to be unpaired. This constraint option has been implemented in Sfold and available through the Sfold web server [32]. However, refolding will cost a hefty computational price. This global model is essentially equivalent to an approach based on exact calculation of ensemble free energies from initial folding and refolding [27]. This approach makes the assumption that the target will re-establish structure equilibrium after siRNA binding. The analysis of siRNA datasets in our study suggests that target cleavage by RNAi machinery appear to be rather rapid so that the target may not have time to refold before cleavage [7]. This issue warrants further investigation.

An extension of the McCaskill algorithm [20] can compute the probability that a block of nucleotides is single stranded [25]. However, for each block, this extension requires re-computation of the partition functions for the entire RNA and is too time consuming to be efficient for scanning through all possible blocks of a long RNA in the search of best target sites. To handle this problem for RNAi application, a short local RNA folding window of size *W *was used, along with *L *and block length *u *[23]. These treatments introduce substantial uncertainty in computational analysis. Indeed, for *u*, the empirically selected optimal values are quite different for two training datasets [23], raising the concern of the general applicability of optimal parameter values learned from one source of data. For a specific mRNA, because it is not possible to have accurate information on its independent folding domains which may be better predicted individually, the overall prediction accuracy would be compromised by a pre-specified local folding window length that does not suit this specific mRNA. The major findings from this study are the same as we previously reported [7], i.e., target accessibility as a down stream factor in the RNAi pathway and duplex asymmetry for facilitating RISC assembly [36,37] are two most important factors for RNAi efficiency. The study also compared predictive performance with other methods, but using only 12 data points from an independent test dataset of 360 siRNAs. There was no comparison involving Δ*G*_disruption_, our energetic parameter for measuring target site accessibility [7]. For a complete comparison of methods, correlation analysis and other statistical analyses such regression with significance assessment would need to be performed for the whole test dataset and preferably other RNAi datasets from different experimental systems.

Clearly, more studies and analyses would be needed to compare these methods and to further investigate relevant issues such as the validity of global or local folding, but such studies and analyses are beyond the focus of this work. Here, we will employ our own parameter, Δ*G*_disruption_, for calculating target site accessibility.

### Other parameters

For measuring the stability of structure of the siRNA guide strand, the minimum free energy (MFE) of the optimal folding was computed with mfold [38]. Because the folding space is rather small for a tiny siRNA, we considered the use of the optimal fold adequate. For the optimal fold of the siRNA guide strand, we computed the number of free-dangling nucleotides (nts) at the 3' end, the number of free-dangling nucleotides (nts) at the 5' end. These numbers of free nts and the number of free 3' nts with at least two free 5' nts were reported to be highly correlated with RNAi activity [15]. For our dataset, there were at least two free 5' nts for 80 of the 101 shRNAs. In addition, the GC % of the siRNA guide strand was computed.

### Structural RNA data set

To estimate frequency of GC in paired or unpaired regions of structural RNAs with secondary structures elucidated from comparative analysis, we considered a representative set of 81 RNA sequences that was used in a previous work [39]. The set included 10 tRNAs, 10 5S rRNAs, 10 RNase P RNAs, 10 SRP RNAs, 10 tmRNAs, nine group I introns, two group II introns, 10 16S rRNAs, 10 23S rRNA.

## Competing interests

The authors declare that they have no competing interests.

## Authors' contributions

CYC, CSC, DDL, AM and YS performed computational analyses. AM generated the shRNA data under the supervision of IR and made the first observation of correlation between GC content and target disruption energy. YD supervised the computational work and wrote the computational portions of the manuscript. IR drafted the description of the shRNA dataset. All authors read and approved the final manuscript.
